# Comparing modelled with self-reported travel time and the used versus the nearest facility: modelling geographic accessibility to family planning outlets in Kenya

**DOI:** 10.1136/bmjgh-2021-008366

**Published:** 2022-05-06

**Authors:** Paul Bouanchaud, Peter M Macharia, Eden G Demise, Doreen Nakimuli, CM4FP Group, Jennifer Anyanti

**Affiliations:** 1 Strategy & Insights, Population Services International, Washington, DC, USA; 2 Population Health Unit, KEMRI-Wellcome Trust Research Programme, Nairobi, Kenya; 3 Centre for Health Informatics, Computing, and Statistics, Lancaster Medical School, Lancaster University, Lancaster, UK; 4 SRH, Population Services International, Washington, DC, USA; 5 Strategic Information & Learning, Population Services International, Kampala, Uganda

**Keywords:** health policy, medical demography, public health, other study design

## Abstract

**Introduction:**

Geographic access to family planning (FP) services has been characterised through a variety of proximity metrics. However, there is little evidence on the validity of women’s self-reported compared with modelled travel time to an FP outlet, or between different distance measures.

**Methods:**

We used data from four urban sites in Kenya. A longitudinal FP outlet census was directly linked with data from cross-sectional FP user surveys. We combined characteristics of outlet visited to obtain FP, transport mode, self-reported travel time and location of households and outlets with data on road networks, elevation, land use and travel barriers within a cost-distance algorithm to compute modelled travel time, route and Euclidean distance between households and outlets. We compared modelled and self-reported travel times, Euclidean and route distances and the use of visited versus nearest facility.

**Results:**

931 contraceptive users were directly linked to their FP source. Self-reported travel times were consistently and significantly higher than modelled times, with greater differences for those using vehicles rather than walking. Modelled and Euclidean distances were similar in the four geographies. 20% of women used their nearest FP outlet while 52% went to their nearest outlet when conditional on it offering their most recently used FP method.

**Conclusion:**

In urban areas with high facility density and good road connectivity, over half of FP users visited their nearest outlet with their chosen method available. In these settings, Euclidean distances were sufficient to characterise geographic proximity; however, reported and modelled travel times differed across all sites.

**WHAT IS ALREADY KNOWN ON THIS TOPIC**

- Geographic access is key for use of family planning (FP) services, but we know that most FP customers bypass their nearest FP outlet in low-resource settings.
- It is expensive to collect geospatial and care-seeking data, and complex to compute robust proximity metrics for geographic access, so most studies rely on the assumption that women use the nearest facility in most geospatial algorithms.
- There is sparse evidence on the validity of women’s self-reported travel time compared with modelled measures of travel time to an FP outlet, and how far women generally travel beyond their nearest outlet to reach their preferred FP source.

**WHAT THIS STUDY ADDS**

- Using a definition of ‘nearest outlet’ that is conditional on it offering an FP user’s chosen method increased the percentage of women who visited their nearest outlet from 20% to 52%, while outlet type did not make much difference.
- Euclidean distance gives very similar results to modelled least cost distances in a small urban to semiurban settings with good road networks and high FP outlet density.
- Modelled and self-reported travel times are significantly different and only moderately correlated.

**HOW THIS STUDY MIGHT AFFECT RESEARCH, PRACTICE AND/OR POLICY**

- There is a need to establish a ‘criterion standard’ approach to better capture and refine self-report questions for studies that are unable to capture geographic data for computing proximity metrics.
- For studies aiming to measure distance between households and other sites, Euclidean distance is probably adequate in urban areas with a high number of outlets and good road network.
- We recommend accounting for the user’s preferred method of FP when identifying ‘nearest FP outlet’ for secondary analyses where this is the assumed outlet visited.

## Background

Access to healthcare, while multidimensional,^1–4^ has geographic accessibility (ie, women’s physical proximity to family planning (FP) outlets) as a key component. Understanding how geographic access affects health service utilisation is central to measures of equity and for healthcare planning globally.^5^ Many studies rely solely on straight-line distance measures to the nearest facility or self-reported travel times to measure geographic accessibility to services, but seldom are these measures compared in the literature, and we know little about how accurately they reflect the experiences of women accessing FP products or services.

Where adequate spatial data on FP care-seeking behaviours have been collected, distance and/or travel time between residential households and outlets may be defined in a number of ways. Two of the most common are Euclidean distance and least cost distance. Euclidean distance is defined as the straight-line distance between two points, which is computationally least demanding, but makes the unrealistic assumption that care-seekers travel in a straight line between their household and the provider. Euclidean distance rarely reflects the reality of movement between a household and an outlet, and often underestimates the distance travelled.^6^ The second measure is least cost distance, which accounts for real-world factors and barriers that affect travel in order to model the most efficient (least expensive) route between two points. This approach estimates travel distance and times based on road networks, land cover, barriers (rivers, water bodies, forests and protected areas), elevation, reported mode of transport and travel speeds. However, the geo-coordinate (both for household and facility used) and geographic feature data needed to calculate distances and travel times can be costly to collect and challenging to analyse, leading to limited evidence of the comparative trade-offs associated with these methods, and therefore limited evidence on the best approach to measuring distance and travel time for seeking healthcare.

Most prior studies of healthcare accessibility have measured distance from a patient’s home to all service delivery points or the nearest outlet within a geographic area, without information on where the patient actually sought care. This indirect matching approach assumes that care was sought at the nearest facility, or that facilities within a specific area may be aggregated to some notional average measure for the area. A systematic review of articles that link households to facility data on reproductive, maternal, newborn and child healthcare found that only 14% (8 out of 59) of articles reviewed had used direct linking/exact matching to the facility where care was sought, while the remainder relied on indirect/ecological linking of households to nearest facilities.^7^

Due in large part to the complexity of gathering accurate geographic information system (GIS) data and modelling least cost routes, most studies have used self-reported travel times or distances. For example, one systematic review identified 57 articles addressing physical access to skilled care for childbirth in sub-Saharan Africa and found that very few studies used GIS data to measure geographic access to care, relying instead on self-report. Among the 40 studies that reported distances, only 38% measured distance using geographic data as opposed to self-reported measures, while only 12% of the studies relying on travel time used clearly defined start and end points (rather than self-report).^8^ The water, sanitation and hygiene literature provides a comparison of self-reported travel times to those estimated using GIS data and suggests that self-reported travel times to water sources were overestimated in one Mozambique study.^6^ Moreover, recent work examining patient travel times for obstetrics^9–11^ and trauma surgery^12^ has suggested that modelled times may underestimate travel times when compared with self-report, while a study among HIV clinic attendees found a low correlation between self-reported and global positioning system (GPS)-based measures of travel time and distance.^13^ The validity and reliability of these self-reported measures are likely to be affected by recall, comprehension and social desirability biases and have rarely been tested.^14^

Although geographic accessibility alone is an insufficient measure, it remains a potentially important determinant of FP use.^15 16^ Existing large-scale surveys such as the Demographic and Health Survey (DHS) and Performance Monitoring for Action surveys that aim to link individuals with nearby facilities provide geographic data that have several limitations. To protect confidentiality, the DHS cluster coordinates are displaced, creating random noise in the data. In addition, such surveys represent a group of households by a single geographic location, which leads to inaccurate proximity estimates. Since users are not directly linked to their source of FP, it is assumed that women go to the nearest facility of a particular type, which introduces further biases in service access measures based on these data.^14 17^ Elewonibi and colleagues’^17^ work in north-eastern Tanzania directly linked FP users to health facilities, but once again relied on Euclidean distances, and furthermore excluded smaller FP outlets, which are an important source of contraception in many communities.

This study contributes to a nascent literature examining the validity of women’s self-reported compared with modelled measures of travel times and distances. Using a novel data set that directly links contraceptive users with a census of FP outlets and identifies which outlet they visited for their current contraceptive, we aim to answer the following questions: (1) How different are straight-line distance measures from least cost routes between households and outlets? (2) How far women travel to obtain FP beyond their nearest facility offering their FP method of choice and by facility type? (3) How well do FP users’ self-reports of time travelled to last source of FP compare to modelled least cost distance? To address our objectives, we compute and compare travel times and distances (both Euclidean and least cost route) travelled by women to their most recently used outlet against women’s self-reported travel times in four geographies in Kenya.

## Methods

This study uses data collected through the Consumer’s Market for Family Planning (CM4FP) project, implemented by Population Services International. The CM4FP project conducted a census of outlets that supply FP products or services in four study sites each in Kenya, Nigeria and Uganda. The sites were purposively selected in urban areas and represent a spectrum of urban areas by size. The analysis presented in this paper uses only data from the four study sites in Kenya. The Kenya data set was selected for analysis here as it had the highest number of directly matched women to FP outlets of the three countries. A full description of the project and its design, including fieldwork dates and other details, is available elsewhere.^18^

### Study sites

The four study sites in Kenya selected include large urban, medium urban, small urban and semiurban areas (within the primary urban settings of Nairobi County, Nakuru County, Kilifi County and Migori County, respectively). Each site was a single ‘ring fenced’ contiguous geographic area. Data were collected from adjoining wards (Kenya’s smallest administrative unit). The study areas within each urban setting were selected based on considerations including health facility density, presence of households in the area, proximity of health facilities to residential areas and a mixture of socioeconomic groups.

### Data

The outlet census was followed by a household survey from an enumeration area located entirely within the outlet census area. Study rounds were repeated on a quarterly basis, with a longitudinal design for the outlet data, and a repeated cross-sectional sample for the household survey. The study design permits direct linkage between recent FP users and their most recently visited outlet—a feature of the data that is central to the analysis we present here. In total, the study visited 664 outlets (including community health workers (CHWs)) and 3816 households in Kenya.

The outlet survey was a longitudinal study of all facilities, outlets and CHWs that provide modern FP commodities or services. These included public health facilities, private facilities (for profit and not for profit), pharmacies, dispensaries, drugstores and mobile CHWs who worked within the community to provide FP products and services. CHWs were excluded from this analysis as they could not be assigned a GPS location. The household data consist of a cross-sectional survey of women who lived within an ‘inner ring’ area inside the outlet census area. This study only presents results on linked data where we were able to link the households to the outlets used and where the woman reported that she travelled from her home to the outlet (rather than her place of work, for example). Direct linking of eligible FP users to their most recently visited FP source was achieved by asking women details about the outlet, such as name, location and personnel names. If the outlet information matched with an outlet included in the census, the FP user was shown a picture of the outlet to confirm the match. Unique IDs were used in the data sets to connect women with their matched outlet.

All the locations of FP outlets and residential households were mapped using GPS hand-held devices. After mapping, all the GPS coordinates were validated by ensuring that the recorded GPS accuracy was acceptable and that points were acceptably consistent between readings, as applicable (such as outlet location between rounds or households within the same or adjacent apartment blocks). Invalid coordinates were substituted from among other available coordinates in the data where possible or were excluded from distance calculations.

In addition, we assembled factors that affect travel including road network, slope based on a digital elevation model (DEM), land use/cover and travel barriers (water bodies). The majority of people travel from their households to seek care along a road network as opposed to travelling in straight lines due to travel barriers; therefore, we assembled spatial layers of the road networks based on existing data sources^19^ and updated them via high-resolution satellite imagery using Google Earth. Roads were classified either as primary, secondary, county or rural roads.^20^ In areas with no road network and spaces between roads, a 2020 land use/cover map derived from European Space Agency (ESA) Sentinel-2 imagery at 10 m spatial resolution was used.^21^ The imagery comprised seven classes within the study area, namely water, trees, grass, flooded vegetation, crops, shrubs and built areas. The water bodies were derived from high-resolution satellite imagery. Finally, Shuttle Radar Topography Mission DEM was available from the Regional Centre for Mapping of Resources for Development online GeoPortal at 30×30 m spatial resolution.^22^

### Distance and time estimation methods

We computed both the travel distance and time between each matched household location in the survey and all FP outlets in the census. In total, 931 FP users were directly matched to an outlet and were included in the analysis. Six hundred and nine outlets were included in the distance and time calculations to women’s household location. In total, there were 117 177 pairs of households and outlets in the analytical data set (each observation consisting of the set of distance/time measures between each FP user and all outlets within a study site). The distance and travel time were calculated based on both the straight-line distance (Euclidean) and route distance (least cost path) (online supplemental table 1).

10.1136/bmjgh-2021-008366.supp1 Supplementary data

### Least cost path travel distance and time

To model realistic travel times and distances, data are needed on mode of transport, route followed, associated speed and which outlet was used. Individual data on the mode of transport (walking, bicycle, motorcycle or vehicular transport) and the location at which the journey was initiated (home or workplace) and ended (specific FP outlet) were available in the CM4FP data.

To model travel times and distances to the nearest or actual FP outlet, AccessMod software alpha V.5.7.8 (WHO, Geneva, Switzerland) was used.^23^ AccessMod uses the terrain-based least cost path distance calculation to model travel time and distances. It has been applied widely in computing spatial access metrics in sub-Saharan Africa.^24–26^ First, we spatially overlaid and merged the road network and land use data to obtain a single raster data set using the ‘merge land cover’ module of AccessMod. Travel speeds based on a review of similar studies^20 24^ shown in online supplemental table 2 were then assigned to each road class and land cover type on the merged surface. Water bodies were regarded as a barrier except in presence of a bridge. We ran three models, each for a specified travel scenario (walking, bicycling or motorised), and assigned each woman the travel time or distance corresponding to their mode of travel.

The walking speeds were adjusted for changes in slope derived from the DEM using Tobler’s hiking function (equation 1), an exponential function that describes how walking speeds vary with slope.^27^ The bicycling speeds were also adjusted for changes in slope based on a bicycling power correction that assumes increased speed due to a negative slope does not exceed twice the speed on flat surfaces.^28 29^ The ‘accessibility module’ of AccessMod was then used to invoke the least cost path algorithm and compute the accumulated travel time from each household to its nearest outlet at 10×10 m spatial resolution:

(1) W=6.exp[−3.5.|s+0.05|]

where: W is the adjusted speed and S is the slope of the terrain derived from the DEM.

To compute the travel time and distance to the actual used outlet we used the ‘referral analysis module’ of AccessMod. The module facilitates the computation of travel times and distances along the least cost path between a set of starting and destination locations. The starting locations were all matched households while the destination locations were all outlets per study site. The algorithm computes these spatial metrics between each pair of starting and destination locations through a double loop. One loop runs through all the starting locations while the second runs through the destination locations. Specifically, the first household was selected, and travel time/distance computed along the least path from this household to all outlets based on a priori defined speeds and merged land cover. The process was repeated until all households had been accounted for and their distances/times to all outlets computed.

### Euclidean distance computation

To compute the straight-line distance between each matched household and its nearest outlet, we used the ‘near’ function of the Proximity toolbox in ArcMap V.10.5 (ESRI, Redlands, California, USA). Further, in the same set-up, the point distance function was used to compute Euclidean distance from each matched household to all the outlets in the study site. The computed Euclidean distance represented the shortest distance, or the ‘as the crow flies’ distance between each matched household and an outlet without factoring in elevation, road network or travel barriers.

All distances (least cost and Euclidean) were rounded to the nearest 10 m after running accessibility modules, prior to further analysis because hand-held GPS receivers are subject to small, expected errors during field observations, such as those due to the satellite array or random errors over which the surveyor has no control. Likewise, GPS readings used for distance calculations include an inherent level of subjectivity (a reading may have been taken outside an outlet, or towards the front or back of an outlet). Finally, two outliers were excluded from the self-reported versus modelled time analysis due to unrealistically long self-reported travel times.

### Defining nearest facility

We defined several types of nearest outlet in our analysis.

- The nearest outlet to the household, not conditional on outlet type or products offered—that is, the nearest outlet of any type, with any methods offered.
- The nearest outlet to the household that offers the FP user’s chosen product (the same product that the user reported obtaining from the matched outlet), not conditional on outlet type.
- The nearest outlet to the household conditional on offering chosen product and matching the type of outlet the user reported visiting.

Wealth quintile was defined using the Equity Tool (www.equitytool.org), and calculated, along with the descriptive analyses presented in the tables using STATA V.15.0 (StataCorp. 2017. *Stata Statistical Software: Release 15*. College Station, Texas: StataCorp). Significance testing for travel time differences was conducted using paired t-tests.

### Patient and public involvement

Patients and the public were not involved in the study.

## Results

### Characteristics of study participants

Across the four study sites in Kenya, 931 women of reproductive age, who were directly linked to the FP outlet where they last sourced their contraceptive method, and who travelled from their home to the outlet were included in the analysis (table 1). Women were eligible for direct matching if they were currently or recently (within 12 months) using a method that they had obtained themselves from an outlet within the study areas. Eighty-four per cent of those eligible for direct matching were successfully matched to an outlet (983 women), and 95% (931) of those women had travelled to the outlet from their home, and so were included in our analysis. The average age of the sample was 29 years. In Kilifi and Migori (the small and semiurban sites, respectively), the women sampled were quite evenly spread across urban wealth quintiles, while those in Nairobi and Nakuru (the large and medium sites, respectively) tended to be in the wealthier quintiles. Education level varied by site, with over half of those in Kilifi and Migori reporting having completed primary education, over half in Nairobi reporting having completed secondary and almost two-thirds of those in the medium urban site reporting postsecondary education. Short-acting methods (male condoms, contraceptive pill/oral contraceptives, and injectables) dominated the method mix in all sites and accounted for over 80% in Nairobi. Implants made up 22% of methods currently or most recently used, while copper intrauterine devices accounted for 1% in the sample across all study sites.

**Table 1:** Description of the directly matched women across four study sites in Kenya

		Large urban (Nairobi)	Medium urban (Nakuru)	Small urban (Kilifi)	Semiurban (Migori)	Total
Matched women (n)		241	149	306	235	931
Age	Mean, years (95% CI)	29.5 (28.7 to 30.4)	28 (27.0 to 29.0)	29 (28.3 to 29.8)	29.3 (28.5 to 30.1)	29 (28.9 to 29.0)
Education, n (%)	No formal	0 (0)	0 (0)	26 (8)	2 (1)	28 (3)
	Completed primary	51 (21)	11 (7)	158 (52)	138 (59)	358 (38)
	Completed secondary	127 (53)	41 (28)	80 (26)	64 (27)	312 (34)
	Higher	63 (26)	97 (65)	42 (14)	31 (13)	233 (25)
Socioeconomic status (urban wealth quintile), n (%)	1	1 (0)	3 (2)	94 (31)	89 (38)	187 (20)
	2	10 (4)	17 (11)	49 (16)	60 (26)	136 (15)
	3	40 (17)	21 (14)	52 (17)	38 (16)	151 (16)
	4	57 (24)	35 (23)	44 (14)	30 (13)	166 (18)
	5	133 (55)	73 (49)	66 (22)	18 (8)	290 (31)
Current/most recent method, n (%)	Male condom	14 (6)	19 (13)	25 (8)	11 (5)	69 (7)
	Contraceptive pill/oral contraceptives	57 (24)	32 (21)	33 (11)	18 (8)	140 (15)
	Injectable	126 (52)	49 (33)	148 (48)	120 (51)	443 (48)
	Implant	36 (15)	26 (17)	71 (23)	75 (32)	208 (22)
	Emergency Contraceptive Pills	4 (2)	18 (12)	24 (8)	8 (3)	54 (6)
	Cu-IUD	4 (2)	5 (3)	3 (1)	1 (0)	13 (1)
	Other/traditional	0 (0)	0 (0)	2 (1)	2 (1)	4 (0)
How did you travel to the matched outlet last time you visited it?, n (%)	Walked	224 (93)	92 (62)	246 (80)	121 (51)	683 (73)
	Bicycle	0 (0)	23 (15)	11 (4)	5 (2)	39 (4)
	Taxi/bus/motorcycle/car	17 (7)	34 (23)	49 (16)	109 (46)	209 (22)

Cu-IUD, copper intrauterine device.

For their last visit to source FP, walking was the most common means of transport used (73% across all sites) the last time FP method was obtained with Nairobi having the highest (93%). However, vehicular transport was reported in a substantial minority of cases (46% and 23%) in Migori (semiurban) and Nakuru (medium urban), respectively.

In total, 609 outlets were matched to FP users. Private sector outlets made up a large majority of all matched outlets (93% across the four sites), and the most common outlet type in all sites was pharmacy/chemist, accounting for between 48% and 62% of all linked outlets in Migori and Nairobi, respectively (table 2).

**Table 2:** Description of the directly matched FP outlet sample across four study sites in Kenya (Nairobi, Nakuru, Kilifi and Migori)

		Large urban (Nairobi)	Medium urban (Nakuru)	Small urban (Kilifi)	Semiurban (Migori)	Total
Matched outlets (n)		223	239	81	66	609
Outlet types, n (%)	Hospital	3 (1)	12 (5)	2 (2)	4 (6)	21 (3)
	Health/medical centre	27 (12)	21 (9)	6 (7)	5 (8)	59 (10)
	Nursing home	0 (0)	3 (1)	1 (1)	4 (6)	8 (1)
	Medical clinic	52 (23)	58 (24)	18 (22)	15 (23)	143 (23)
	Pharmacy/chemist	139 (62)	137 (57)	41 (51)	32 (48)	349 (57)
	Dispensary	1 (0)	6 (3)	11 (14)	6 (9)	24 (4)
	Other	1 (0)	2 (1)	2 (2)	0 (0)	5 (1)
Managing authority, n (%)	Public/government	11 (5)	12 (5)	10 (12)	6 (9)	39 (6)
	Private	211 (95)	222 (93)	71 (88)	60 (91)	564 (93)
	Others	1 (0)	5 (2)	0 (0)	0 (0)	6 (1)

FP, family planning.

The percentage of FP users whose matched outlet was their nearest outlet shows little variation according to which measure of distance or time is chosen. Focusing on Euclidean distance, across all sites just under 20% of FP users visited their nearest outlet (table 3). When nearest outlet included the condition of offering the user’s chosen FP method, 52% of users went to their nearest outlet. When the definition of nearest outlet was further restricted to be conditional on both product and outlet type, we see a small increase in the percentage of FP users who went to their nearest outlet (54% overall). For all three definitions of nearest outlet, higher levels of visiting the nearest outlet were seen in the smaller compared with larger urban sites.

**Table 3:** Percentage of matched FP users who visited nearest facility disaggregated across three domains (any nearest facility, nearest facility conditional on chosen product, nearest based on product and outlet type)

	Large urban (Nairobi)	Medium urban (Nakuru)	Small urban (Kilifi)	Semiurban (Migori)	Total
% of matched FP users visiting their nearest outlet, not conditional on outlet type or product availability					
Euclidean distance	24.5	17.4	15.7	21.7	19.8
Modelled least cost distance	24.5	17.4	15.4	20.9	19.4
% of users who visited nearest outlet of any type offering their chosen product defined by:					
Modelled least cost distance	43.6	41.6	66.0	49.8	52.2
% of users who visited nearest outlet of their chosen type, offering their chosen product, defined by:					
Modelled least cost distance	44.0	41.6	66.7	54.0	53.6

FP, family planning.

For all distance and time measures, the difference between distance to nearest outlet and outlet visited was quite small in all sites (table 4). This difference in distances between actual outlet visited and nearest outlet (of chosen type, offering chosen method) was higher in Migori (the geographically largest site) than the other three sites. While modelled least cost difference inevitably shows slightly larger distances than the Euclidean measure, the magnitude of difference between outlet visited and nearest outlet was very similar between these two measures.

**Table 4:** Distance and travel time to the nearest outlet versus the actual outlet visited, by modelled distance measure

		Large urban (Nairobi)	Medium urban (Nakuru)	Small urban (Kilifi)	Semiurban (Migori)	Total
Euclidean distances (km)	Mean distance to nearest outlet of same type offering method	0.33	0.53	0.68	1.25	0.71
	Mean distance to visited outlet	0.5	0.99	0.91	1.93	1.07
Modelled least cost distances (km)	Mean distance to nearest outlet of same type offering method	0.35	0.56	0.74	1.34	0.76
	Mean distance to visited outlet	0.53	1.05	0.98	2.07	1.15
Modelled travel times (min:s)	Mean time to nearest outlet of same type offering method	3:54	4:36	6:42	9:48	6:24
	Mean time to visited outlet	5:42	7:30	8:48	11:42	8:30
	Mean time to visited outlet (among those going to nearest)	3:54	5:24	6:18	9:54	6:30
	Mean time to visited outlet (among those not going to nearest)	7:24	8:54	13:24	13:24	10:42
**Self-report and modelled time differences, by transport type**						
Mean time to visited outlet (walked)	Self-reported travel time (min:s)	14:05	11:54	14:41	20.08	15:05
	Modelled travel times (min:s)	6:03	9:04	10:00	14.11	9:19
	Difference	8:02***	2:50*	4:41***	5:57***	5:47***
Mean time to visited outlet (other means of transport)	Self-reported travel time (min:s)	12:25	10:24	15:20	16:38	14:35
	Modelled travel times (min:s)	1:28	4:59	3:34	9:02	6:16
	Difference	10:57***	5:25***	11:46***	7:36***	8:19***

Significance levels: *p<0.05; **p<0.01; ***p<0.001.

**Figure 1:**
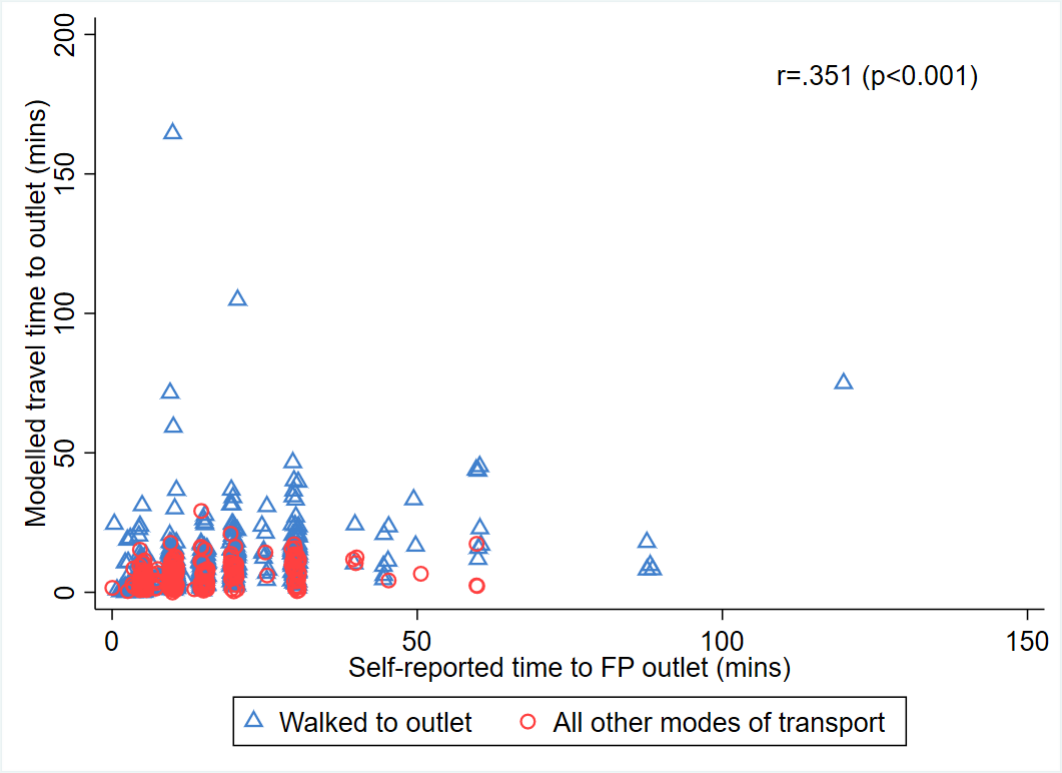
Scatter plot showing correlation between modelled and self-reported travel times, by mode of transportation. FP, family planning.

When difference in travel time is estimated, we see similar time differences across all sites (of around 2 min): on average, FP users travelled 1–2 min further to their chosen outlet than if they went to their nearest outlet of the same type that offers their chosen method. However, when we disaggregate travel times for those who did and did not go to their nearest outlet, we find the latter group are in some cases travelling for a considerably longer time to access FP. In Kilifi, for example, there is a 5 min difference (equivalent to more than doubling the travel time) between those who went to their nearest outlet (6 min) and those who did not (13 min). Further comparisons of those who did and did not go to their nearest outlet are shown in supplemental table 3. Table 4 also compares self-report with modelled travel times for both FP users who reported walking and those who used other transport to their matched outlet to access their most recent FP method. Across all four sites, self-reported travel times were longer than modelled travel times, but with a smaller discrepancy for those who walked to the outlet compared with other means of transport. For those walking to an outlet, the difference between self-reported and modelled travel time was almost 6 min overall, while for those using other means of transport the difference was over 8 min. All differences were statistically significant at the 5% level. Overall, figure 1 shows the distributions of measures of self-report and modelled travel times. The Pearson correlation coefficients for self-report and modelled times were 0.367 (p<0.001), 0.314 (p<0.001) and 0.351 (p<0.001) for walking, other means of transport and all methods, respectively.

## Discussion

Using a rich source of survey data with directly linked household and outlet data alongside a range of proximity metrics, our analyses contribute to an improved understanding of how different measures of geographic access compare in the context of accessing FP products. Our analyses showed that reported travel times were significantly higher than modelled travel times, even in small geographic settings, and that there is utility of accounting for FP method type when computing travel time to the nearest FP outlet. In denser urban settings with a good spatial distribution of FP outlets and improved road networks, the differences between Euclidean and route distance were small.

Using directly matched households and outlets allowed us to compare modelled and self-reported travel times and we found that self-report appeared to be systematically longer than the modelled times, with a moderate correlation, similar to that found elsewhere.^10^ The difference was particularly marked among those reporting non-walking means of transport, while those walking in Nairobi (who, unlike their vehicle-using peers, are not subject to traffic jams) had relatively large differences between modelled and self-reported times. These findings raise questions about the assumptions of the models for these types of journeys, as well as the potential for recall bias in self-report.^6^ In the self-report data, we saw some evidence of heaping or rounding off around certain times (10, 20, 30 min). Modelled times do not account for traffic, waiting times, uncertainty in the travel speeds, and so on, which may also have led to some of the differences seen. Accurate travel time estimation is an important element for understanding access to FP and other health services as it represents objective measure of accessibility.^24 30^ However, self-reported travel times remain critical as they provide insight into the user’s experience or perceptions of access. Future research into the accuracy of self-report versus modelled travel times might consider the use of timers for journeys or GPS tags to gather data on the actual routes taken to FP outlets. Recently, this was achieved by replicating journeys of women by professional drivers^9^; however, it might be expensive for a study with many participants. Further, the travel conditions that were prevailing when women made their journeys might be different from those encountered by the professional drivers.

Our results also show that by using directly matched data, we can go further than many established approaches that assume FP users go to their nearest outlet. Elewonibi and colleagues’^17^ work found that 33% of FP users obtained their method from the nearest outlet. The CM4FP data covered the total market for FP products, including smaller private sector outlets like chemists and pharmacies that other studies have tended to ignore, but that make up a large number of FP outlets in many sites. This inclusion resulted in even fewer women identified as going to their nearest outlet (20% across the study sites). We further found that this estimate held for both Euclidean and least cost distance and time estimates. This underlines the importance of better understanding FP users’ range of reasons for selecting an outlet. These factors likely include reputation or perceived service quality, cost, the convenience of other amenities along the route and, of course, personal preferences,^31 32^ particularly when the difference in distance or travel time between the nearest and actually visited outlet is quite small.

Our findings also have important implications for understanding bypassing behaviour for FP use. We found that the proportion of FP users who went to their nearest outlet showed considerable variation according to how the ‘nearest outlet’ was defined. If this is purely the geographically closest outlet to the household, then only about one in five users visited their nearest outlet. However, if the definition of the nearest outlet was conditional on the outlet having the FP user’s chosen method available, over half of the directly linked FP users went to their nearest outlet. Making the definition of nearest outlet also conditional on outlet type, however, made very little difference to the proportion of users found to have visited their nearest outlet. Other studies that must rely on indirect matching methods between FP users and outlets might consider using FP method availability, rather than outlet type, when indirectly linking users to their FP service environment.

The geospatial data in the CM4FP data set allowed us to measure and compare modelled least cost travel times and distances, self-reported travel times and Euclidean distances between households and FP outlets. We found that Euclidean distance measures, despite being full of assumptions and generally underestimating the consumer’s actual distance to a facility, gave very similar results to modelled least cost distances in these sites. In these smaller predominantly urban sites, this suggests that Euclidean distances may be sufficient for all but the most precise geospatial analyses examining supply-side and demand-side FP market interactions.

### Limitations

This paper focused on FP users who were linked directly to an outlet, and who travelled to the outlet from home. Therefore, we cannot draw any conclusions about those travelling to FP outlets from other places (who may be systematically different). Our analyses were limited by small-sized study areas with a high density of facilities within urban areas. The study design is non-representative of higher geographic or administrative areas, and results from the study are not intended to be generalisable to a wider population. Therefore, we cannot extend the findings nationally or to larger geographic areas within Kenya. However, our approaches can be replicated in the rural areas and areas in the rural-urban continuum to generate similar insights that might allow generalisation across Kenya and similar countries in low-resource settings.

In computing the travel times and distances, we were not able to account for weather and seasonal variations in the study area, in part because the data collection did not cover a full year. Severe weather conditions such as flooding may impact accessibility to FP outlets, especially on non-tarmacked roads.^25 33^ We were also not able to capture variations in speeds due to traffic jams^34^ or other travel barriers that vary by time of day. Therefore, our estimates represent an optimistic scenario where there are minimal traffic jams and no adverse weather conditions. Additionally, most FP users used a walking mode of transport and did not report any adverse event that affected their travel when accessing an FP outlet. The travel times and distance presented represent a one-way journey from a household to an FP outlet and were not adjusted for return journeys nor for any waiting time at the FP outlet.

The speeds used in the accessibility models have important consequences on the correlation between modelled travel times and reported travel times.^10^ Here, speeds were derived from studies in similar contexts^16 20^ and align with recent literature.^11^ This was necessary due to lack of data in our study setting describing the travel speeds that FP users use for each mode of transport. Individuals are unique and might walk, drive, cycle at different paces. However, our approach accounts for the actual mode of transport that was used by FP users, which increases the preciseness of our results, rarely the case in previous studies. Irrespective, going forward, it will be essential to have exemplar observational studies across sub-Saharan Africa that will derive fairly generalisable travel speeds across different road types, land classes, traffic and weather conditions that can be applied across a broad range of applications given the resource constraints of detailed travel specificity data in low-resource settings. Some of the recent and alternative approaches have made use of Google Maps and extracted travel times at time and day that a journey was made, potentially accounting for weather and traffic conditions.^9^ This approach, although still nascent, has potential in revolutionising estimation of ‘closer to reality’ travel times in low-resource setting.

## Conclusion

Here we have used a rich data source directly identifying which FP outlet was used, the mode of transport, where the journey started and the self-reported travel time. We used these data sets and other geospatial factors to describe a broad range of spatial access metrics to FP services in four urban or semiurban sites in Kenya. The results show that modelled and self-reported travel times were significantly different and only moderately correlated. Consequently, the utility of self-reported travel times requires further investigation and standardising the way self-reported times are measured. We also show there was not much difference between Euclidean and least cost route distance (the latter accounting for factors that affect travel). Further, while only a small percentage of users had visited their nearest FP outlet (about 20%), when we made nearest outlet conditional on outlets having the chosen FP product available, over half of FP users had visited their nearest outlet to obtain their method. These findings are key for policymakers to increase uptake of FP and in shaping geographic access to methods.

## Data Availability

Data are available upon reasonable request. All data and documentation for the CM4FP project are available through the project website (www.cm4fp.org).
