# Supplementary material for: Comparing modelled with self-reported travel time and the used versus the nearest facility: modelling geographic accessibility to family planning outlets in Kenya
Source: BMJ Glob Health. 2022 May 6;7(5):e008366. doi: 10.1136/bmjgh-2021-008366 (PMC9083396; doi:10.1136/bmjgh-2021-008366)
Supplement: Supplementary data [file bmjgh-2021-008366supp001.pdf]

Supplemental materials

Supplemental Table 1: A matrix of travel time, distances between matched household locations and family planning service providers either empirically computed or reported by the users of these outlets

	**Distance**	**Time**
**Straight line (Euclidean)**	All outlets in study site	-
**Route (least cost path)**	All outlets in study site	All outlets in study site
**Self-reported**	-	Directly linked (utilized) outlet

Supplemental Table 2: Average travel speeds (km/h) across different land cover and road types in urban areas derived from existing literature

Road and land cover	Walking	Bicycling	motorized
Tree Cover	3.5	7	5
Shrub Cover	4	8	10
Grassland	3.5	7	10
Cropland	4.5	9	5
Built Up Areas	5	11	20
Open Water and regularly flooded areas	0	0	0
Primary road	5	11	70
Secondary road	5	11	50
County road	5	11	45
Rural road	5	11	30

Supplemental analysis: comparing characteristics of those going or not going to their nearest outlet

We compared those who did and did not go to their nearest FP outlet (of the same type with their chosen product available) (Supplemental table 3). We found those who went to their nearest outlet were significantly more likely to have walked in all sites and were significantly less wealthy (in Narobi and Kilifi) than those who bypassed their nearest outlet. No differences were seen in choice of public or private sector outlet, or in use of long-acting reversible contraceptive (LARC) methods.

Supplemental table 3: Characteristics of users by use of nearest facility (time taken, least cost)

	Large Urban (Nairobi)		Medium Urban (Nakuru)		Small Urban (Kilifi)		Semi-Urban (Migori)		**Total**	
N	241		149		306		235		931	
N of FP users going to nearest outlet	108		61		195		103		467	
**Did the user visit the nearest outlet?**	No (n = 108)	Yes	No	Yes	No	Yes	No	Yes	No	Yes
% LARC users	18.0%	14.8%	21.6%	19.7%	22.5%	25.1%	33.3%	31.1%	24.1%	23.3%
% Managing authority: Private sector	90.1%	96.9%	92.9%	94.1%	87.7%	87.6%	89.0%	90.7%	91.2%	92.0%
% Walked to facility (vs. all other transport)	89.5%	97.2%	47.7%	82.0%	66.7%	88.2%	28.8%	80.6%	58.8%	87.8%
Mean wealth quintile	4.4	4.2	4.2	3.9	3.1	2.7	2.4	2.1	3.5	3.0

Note: LARC = long-acting reversible contraception
